# RNA干扰GRP75表达改善人肺腺癌细胞对顺铂耐药性

**DOI:** 10.3779/j.issn.1009-3419.2011.04.01

**Published:** 2011-04-20

**Authors:** 思恩 石, 泽锋 何, 建春 蔡, 江锋 邱

**Affiliations:** 1 361003 厦门，厦门大学附属第一医院肿瘤外科 Department of Tumor Surgery, First Affiliated Hospital of Xiamen University, Xiamen 361003, China; 2 361004 厦门，厦门大学附属中山医院胸外科 Department of Thoracic Surgery, Zhongshan Affiliated Hospital of Xiamen University, Xiamen 361004, China

**Keywords:** 肺肿瘤, 顺铂, GRP75, 耐药性, RNA干扰, 慢病毒, Lung neoplasms, Cisplatin, GRP75, Drug resistance, RNA interference, Lentivirus

## Abstract

**背景与目的:**

GRP75（glucose regulated protein 75）属于热休克蛋白（hot shock protein, HSP）家族，是一种主要位于线粒体的分子伴侣，在某些耐药的肿瘤细胞中高表达。本研究通过慢病毒介导的RNA干扰技术沉默*GRP75*基因的表达，观察下调GRP75的表达对肿瘤细胞耐药性的影响并探讨GRP75在肿瘤细胞耐药机制中的作用。

**方法:**

以顺铂为诱导药物，人肺腺癌细胞系A549为诱导对象，采用逐步增加剂量与大剂量冲击相结合的方法，诱导建立耐顺铂细胞株A549/CDDP。分别将包裹GRP75-shRNA和不含干扰序列的慢病毒转染A549和A549/CDDP细胞，在荧光显微镜下观察各组细胞转染率，应用MTT比色法检测干扰前后各组细胞对顺铂的敏感性，应用Western blot检测干扰前后各组细胞GRP75、p53、bcl-2的表达。

**结果:**

各组细胞感染效率均在90%以上，转染后A549/CDDP和A549细胞中GRP75的表达均明显下调（*P* < 0.05）。转染前后A549/CDDP细胞对顺铂的耐药指数分别为21.52和4.14。转染后A549/CDDP细胞内p53的表达上调（*P* < 0.05），bcl-2表达下调（*P* < 0.05）。

**结论:**

GRP75是A549细胞对顺铂耐药机制的相关蛋白之一，其在耐药机制中的作用与其对p53和bcl-2的调控有关。

## Abstract (en)

**Background and objective:**

GRP75, a member of HSPs which is overexpressed in some resistant cancer cells, is a molecular chaperone mainly located in mitochondrial membrane. The aim of this study is to investigate the role of GRP75 on the resistant mechanisms of cancer cells by downregulating GRP75 expreesion via RNAi approach.

**Methods:**

Cisplatin-resistant cell A549/CDDP was established from their parental human lung adenocarcinoma cell line A549 by combining gradually increasing concentrations of cisplatin with high dosage impact. The shRNA for GRP75 was transfected into A549 and A549/CDDP cells by lentivirus. Western blot and methyl thiazolyl tetrazolium (MTT) assay were applied to detect the influence of silencing GRP75 expression on sensitivity of the cells to cisplatin.

**Results:**

The infection rate of six groups were all over 90%. After infection, the level of expression of GRP75 in both A549 and A549/CDDP were down-regulated (*P* < 0.05); the level of expression of p53 in A549/CDDP was up-regulated (*P* < 0.05) and the level of expression of bcl-2 of A549/CDDP was down-regulated (*P* < 0.05). The resistance index of A549/CDDP before and after infection were 21.52 and 4.14 respectively.

**Conclusion:**

Cisplatin resistance of lung cancer cells is associated with overexpression of *GRP75* gene, which could regulate the expressions of p53 and bcl-2.

目前肺癌已经成为我国发病率和死亡率最高的恶性肿瘤之一，大约70%的患者在诊断时已发展至晚期，无法通过手术治愈，故化疗成为肺癌综合治疗中的重要部分。但是肿瘤细胞对化疗药物发生耐药常导致化疗失败。近年来，人们对肿瘤细胞的耐药机制进行了深入的研究。我们前期的研究采用蛋白质组学方法，研究人肺腺癌细胞A549对顺铂发生耐药前后的蛋白质表达变化，观察到GRP75在耐药细胞中高表达^[1]^。在本次研究中，我们通过慢病毒介导的RNA干扰技术沉默耐药细胞中*GRP75*基因的表达，观察干扰前后肿瘤细胞对顺铂敏感性的变化，探讨GRP75在肺癌细胞耐药机制中的作用，以期为肺癌的治疗发现新的靶点，为揭示肺癌的耐药机制提供线索，并为寻找逆转耐药途径提供基础。

## 1 材料与方法

### 1.1 材料

人肺癌细胞系A549购自中国典型培养物保藏中心（China Center for Type Culture Collection, CCTCC），A549/CDDP细胞为前期研究中诱导建立^[1]^。主要试剂包括：HyQ RPMI-1640培养液（Sigma公司）、10%优级新生牛血清（Gibco公司）、GRP75 shRNA (h) Lentiviral Particles（Santa Cruz公司）、Control shRNA Lentiviral Particles（Santa Cruz公司）、Polybrene（Santa Cruz公司）、兔抗人GRP75多克隆抗体（Cell Signaling公司）、兔抗人bcl-2单克隆抗体（Cell Signaling公司）、兔抗人p53单克隆抗体（Cell Signaling公司）、兔抗人β-actin多克隆抗体（Cell Signaling公司）、胰蛋白酶（1:250，Gibco公司分装）、顺铂（CDDP，Sigma公司）、DMSO试剂级（Amresco公司）、噻唑蓝（MT，Ultra Pure Grade，Amresco公司）、碘化吡啶PI（Sigma公司）、Rnase（Sigma公司）、国产分析纯。主要仪器包括：SW-CJ-ZFD型双人单面超净工作台（苏州净化设备有限公司）、Forma Series Ⅱ Wcta Jacketed CO_2_ Incubator水套培养箱（Thermo Electron Corporation）、DK-S24型电热恒温水浴锅（上海精密实验设备有限公司）、高速离心机（日立公司）、荧光显微镜（奥林帕斯公司）、Touch Screen F039300酶标仪（Sunrise Remote）。

### 1.2 方法

#### 1.2.1 细胞培养

将A549和A549/CDDP细胞培养于含10%新生小牛血清、200 μg/mL庆大霉素的RPMI-1640培养基中，置于37 ℃、5%CO_2_孵箱中，在饱和湿度下培养，隔日换液。每4-5天以1:3的比例用0.25%胰蛋白酶消化传代1次，调整细胞密度不超过5×10^5^个/mL。

#### 1.2.2 慢病毒转染A549细胞

将1×10^4^个A549细胞接种于24孔板，加入1 mL新鲜培养液，培养生长24 h，当50%以上细胞贴壁后开始进行转染。配制培养液和Polybrene的混合液，混合液中Polybrene浓度为5 μg/mL。室温下充分解冻GRP 75 shRNA (h) Lentiviral Particles，并轻摇使之均匀。弃去孔中液体，加入1 mL混合液和100 μL慢病毒，轻轻旋转12孔板使之混匀，培养生长24 h后，弃去孔中液体，将孔内细胞转移至培养瓶中进行常规培养传代，将此组细胞命名为A549/I。按上述相同方法，将Control shRNA Lentiviral Particles转染A549细胞，将转染后的细胞命名为A549/C。

#### 1.2.3 慢病毒转染A549/CDDP细胞

按1.2.2所述方法，将GRP 75 shRNA (h) Lentiviral Particles转染A549/CDDP细胞，将转染后的细胞命名为CDDP/I；将Control shRNA Lentiviral Particles转染A549/CDDP细胞，将转染后的细胞命名为CDDP/C。

#### 1.2.4 荧光显微镜观察

以上4组细胞A549/I、A549/C、CDDP/I、CDDP/C，分别于转染后24 h、48 h、72 h后于荧光显微镜下观察细胞转染情况。

#### 1.2.5 Western blot检测各组细胞GRP75、p53、bcl-2表达

转染后7天，取A549、A549/I、A549/C、A549/CDDP、CDDP/I、CDDP/C六组细胞各一瓶，弃去培养基，以PBS轻柔洗去残余培养基，加入RIPA裂解液吹打细胞，转移至EP管冰浴，离心后收取上清。然后BCA测定各组蛋白浓度，每组取20 μg蛋白。将各组收集的蛋白样品与2×SDS-PAGE蛋白上样缓冲液以1:1混合，置于沸水浴加热5 min，以充分变性蛋白，后置于冰上2 min以冷却至室温，行12%SDS-PAGE电泳，预计目的蛋白已经被适当分离后停止电泳。电泳完毕后，切胶，使用Bio-Rad的标准湿式转膜装置，设定转膜电流为300 mA-400 mA，转膜时间为30 min。转膜完毕后，立即把蛋白膜放置到洗涤液中漂洗2 min，以洗去膜上的转膜液。吸尽洗涤液后，加入20 mL含5%脱脂奶粉的PBST封闭液，室温下封闭1 h，弃去封闭液。加入按1:500稀释的兔抗人GRP75多克隆抗体，加入β-actin作为内参，4 ℃孵育过夜，洗涤液洗膜3次，吸尽洗涤液。加入按1:4, 000稀释的、辣根过氧化物酶（HRP）标记的抗兔二抗，4 ℃孵育1 h，洗涤液洗膜3次，吸尽洗涤液。蛋白检测：加入BeyoECL Plus显色剂，X片曝光，显影，定影。按以上同样方法检测各组细胞p53和bcl-2蛋白表达。

#### 1.2.6 MTT法检测各组细胞顺铂敏感性

分别取A549、A549/I、A549/C、A549/CDDP、CDDP/I、CDDP/C六组细胞各一瓶，用含10%小牛血清的RPMI-1640培养基制成单细胞悬液，以5×10^3^个/孔接种于96孔培养板，每孔体积200 μL，置于37 ℃、饱和湿度、5%CO_2_培养箱中培养。24 h后，更换培养液，按组分别加入200 μL含不同浓度顺铂（0、0.025 μg/mL、0.05 μg/mL、0.1 μg/mL、0.2 μg/mL、0.4 μg/mL、0.8 μg/mL、1.6 μg/mL、3.2 μg/mL、6.4 μg/mL、12.8 μg/mL）的培养基。每个药物浓度设6个复孔，并取不加细胞仅加培养基孔作为空白对照组，接种细胞但不加药物孔作为对照组。48 h后，吸取孔内液体，PBS清洗2次，每孔加入5 mg/mL MTT溶液20 μL，温箱中培养4 h。然后吸取孔内液体，每孔加入DMSO 150 μL，振荡10 min后，选择530 nm波长，在酶联免疫检测仪上测定各孔光吸收值（optical demsity, OD）。根据每个药物浓度组OD值（平均值）计算细胞存活率（vital rate, VR）：VR（%）=（用药组OD值-空白对照组OD值）/（对照组OD值-空白对照组OD值）×100%。通过改良寇式法计算药物的半数抑制浓度（50% inhibitory concentration, IC_50_），公式为：lgIC_50_=Xm-Ix[P-(3-Pm-Pn)/4]。其中Xm=lg最大剂量，I=lg（最大剂量/相临剂量），P=阳性反应率之和，Pm=最大阳性反应率，Pn=最小阳性反应率。之后计算各组细胞耐药指数（resistance index, RI），公式为：RI=检测组IC_50_/A549组IC_50_。

#### 1.2.7 统计学分析

应用统计软件SPSS 12.0进行数据分析，组间差异分析采用*t*检验，以*P* < 0.05为差异具有统计学意义。

## 2 结果

### 2.1 荧光显微镜观察转染效率

感染慢病毒的细胞在荧光倒置显微镜下可以观察到绿色荧光，计数发出绿色荧光的细胞数，其占总细胞数的比例即为感染效率，荧光显微镜显示在48 h各组感染效率均已达到90%以上（图 1）。

**1:**
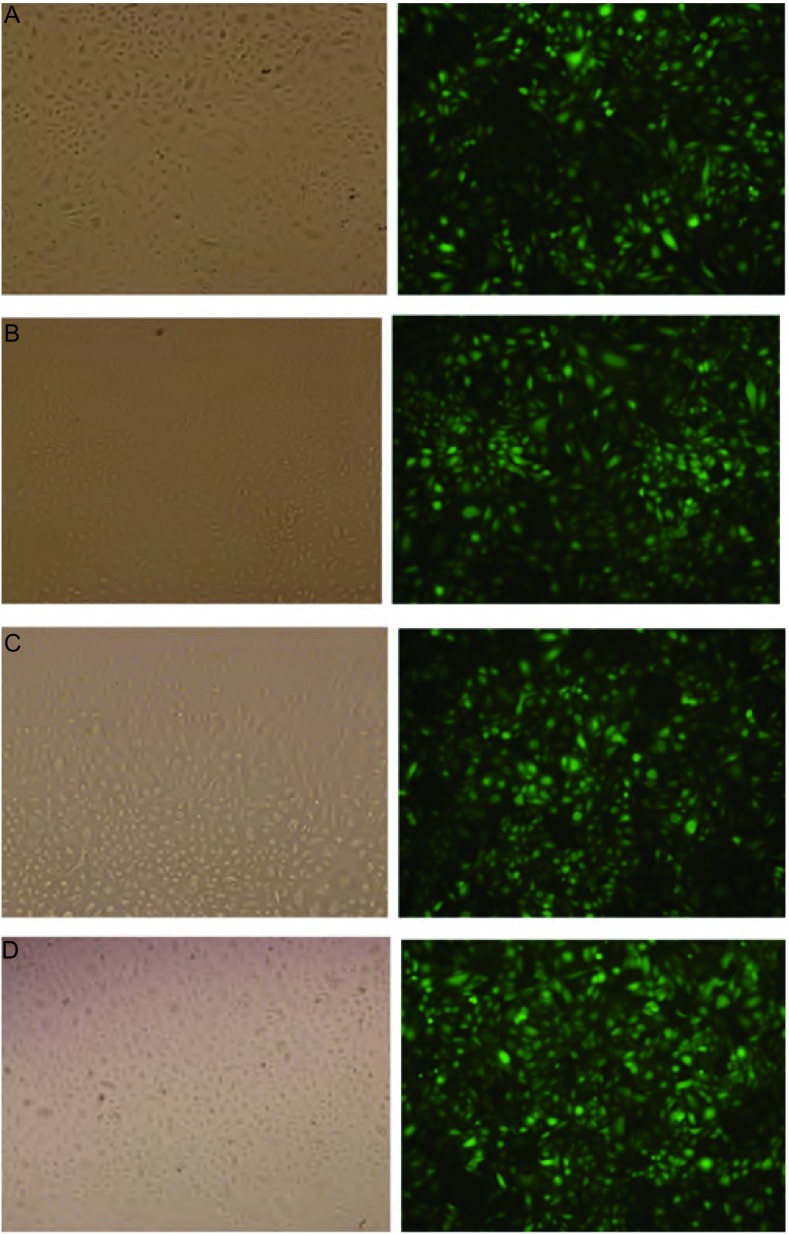
慢病毒载体转染48 h A549细胞和A549/CDDP细胞荧光图（×100）。A：A549/I；B：A549/C；C：CDDP/I；D：CDDP/C。左为明场，右为暗场。转染率为暗场中发绿色荧光的细胞数与明场中细胞总数的比值。可以观察到，各组的转染率均在90%以上。 Fluorography of A549 cells and A549/CDDP cells at 48 h after transfection (×100). A: A549/I; B: A549/C; C: CDDP/I; D: CDDP/C. Bright fields on the left and dark fields on the right. Transfection rate is calculated as ratio between green-fluorescent cell count in the dark field and cell count in the bright field. The transfection rates of 4 groups are all above 90%.

### 2.2 转染后各组细胞GRP75蛋白表达

Western blot检测显示在慢病毒RNA干扰7 d后，各组细胞中的GRP75蛋白表达。实验显示，携带GRP75 shRNA的慢病毒干扰A549细胞后，A549细胞中的GRP75表达明显下调（*P* < 0.05），而不含干扰序列的慢病毒干扰A549细胞后，A549细胞中的GRP75表达无明显变化（*P* > 0.05）。携带GRP75 shRNA的慢病毒干扰A549/CDDP细胞后，A549/CDDP细胞中的GRP75表达明显下调（*P* < 0.05），而不含干扰序列的慢病毒干扰A549/CDDP细胞后，A549/CDDP细胞中的GRP75表达无明显变化（*P* > 0.05）。（图 2A，图 2D）。

**2:**
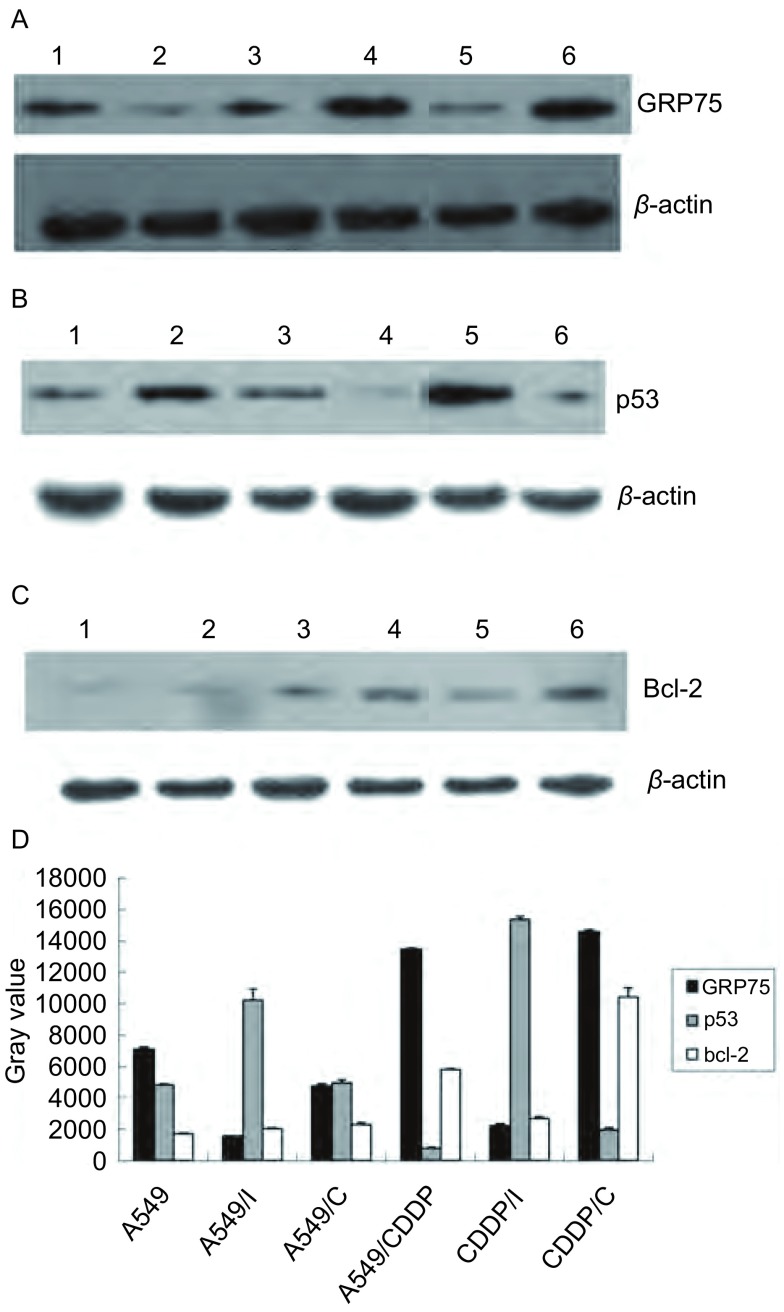
Western blot检测各组细胞GRP75、p53以及bcl-2的表达。A：GRP75；B：p53；C：bcl-2；D：各组细胞GRP75、p53、bcl-2相对表达量。 Western blot assay of GRP75, p53 and bcl-2. A: GRP75; B: p53; C: bcl-2; D: Relative quantitative of GRP75, p53, bcl-2 in six groups of cells. 1: A549; 2: A549/I; 3: A549/C; 4: A549/CDDP; 5: CDDP/I; 6: CDDP/C.

### 2.3 转染后各组细胞对顺铂敏感性

图 3显示的是不同浓度的顺铂作用48 h后，A549细胞、A549/I细胞、A549/C细胞、A549/CDDP细胞、CDDP/I细胞、CDDP/C细胞生长受到抑制的情况。在细胞存活率方面，相同浓度顺铂下，A549/I *vs* A549、CDDP/I *vs* A549/CDDP均下降（*P* < 0.05），CDDP/I的下降幅度大于A549/I（*P* < 0.05）；而A549/C *vs* A549、CDDP/C *vs* A549/CDDP的存活率则无明显差异（*P* > 0.05）。表 1显示经过MTT检验计算后的各组细胞对顺铂的IC_50_和耐药指数。结果显示，在IC_50_方面，A549/I *vs* A549、CDDP/I *vs* A549/CDDP下降（*P* < 0.05），但CDDP/I的IC 50仍然高于A549，而A549/C *vs* A549、CDDP/C *vs* A549/CDDP则无明显差异（*P* > 0.05)。在耐药指数方面，A549/I *vs* A549、CDDP/I *vs* A549/CDDP下降（*P* < 0.05），但CDDP/I的耐药指数仍然高于A549，而A549/C *vs* A549、CDDP/C *vs* A549/CDDP则无明显差异（*P* > 0.05）。

**3:**
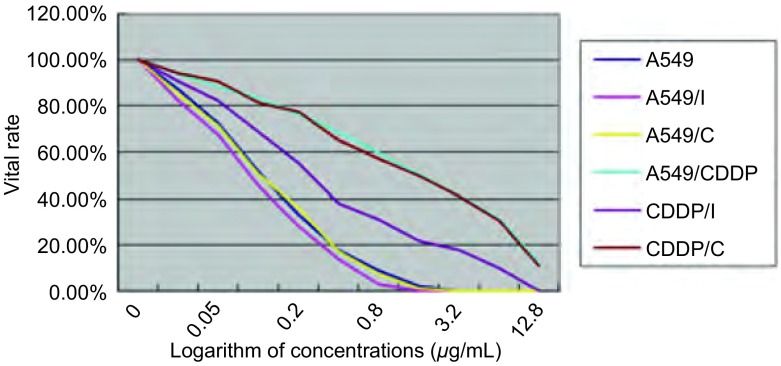
不同浓度顺铂作用48 h各组细胞存活率 48 h vital rate of 6 groups of cell in different concentrations of cisplatin

**1:** 各组细胞对顺铂IC_50_及耐药指数 IC_50_ and RI of six groups of cells to cisplatin

Cell	IC_50_ (*μ*g/mL)	Resistance index
A549	0.072±0.008	1.00
A549/I	0.036±0.008	0.5
A549/C	0.07±0.010	0.97
A549/CDDP	1.55±0.034	21.52
CDDP/I	0.298±0.029	4.14
CDDP/C	1.52±0.037	21.11

### 2.4 慢病毒转染后各组细胞p53蛋白表达

Western blot检测显示在慢病毒RNA干扰后，各组细胞中的p53蛋白表达。实验显示，A549/CDDP细胞的p53表达较A549细胞明显下调（*P* < 0.05）；A549细胞经慢病毒RNA干扰后p53表达明显上调（*P* < 0.05），而经空病毒干扰后p53表达轻微上调（*P* < 0.05)；A549/CDDP经慢病毒RNA干扰后p53表达明显上调（*P* < 0.01），而经空病毒干扰后p53表达轻微上调（*P* < 0.05）（图 2B，图 2D）。

### 2.5 转染后各组细胞bcl-2蛋白表达

Western blot检测显示在慢病毒RNA干扰后，各组细胞中的p53蛋白表达。实验显示，A549/CDDP细胞的bcl-2较A549细胞明显上调（*P* < 0.05）；A549细胞经慢病毒RNA干扰后bcl-2表达无明显改变（*P* > 0.05），而经空病毒干扰后bcl-2表达上调（*P* < 0.05）；A549/CDDP经慢病毒RNA干扰后p53表达明显下调（*P* < 0.01），而经空病毒干扰后p53表达轻微上调（*P* < 0.05）（图 2C，图 2D）。

## 3 讨论

近年来，RNAi作为一种实验技术，在关于肿瘤细胞耐药机制，以及逆转肿瘤细胞耐药方法的研究中，表现出令人欣喜的成效。Wu等^[2]^通过siRNA有效地抑制肿瘤细胞中MDR1的mRNA和P-gp的表达。July等^[3]^设计的凝聚素siRNA可明显抑制凝聚素的表达，从而增强了体外肿瘤细胞对化疗药物的敏感性。Yuan等^[4]^将mdr1 siRNA转染多药耐药乳腺癌细胞MCF-7/Adr后证实特异性siRNA能明显提高阿霉素对MCF-7/Adr细胞的杀伤作用，逆转细胞耐药性。通过RNAi技术抑制肿瘤细胞耐药相关基因的表达，正在成为克服肿瘤细胞耐药的新策略，它为逆转肿瘤耐药提供了一种新的思路，有较好的应用前景。

本研究采用携带GRP75 shRNA的慢病毒进行RNA干扰。在RNA干扰后48 h，通过荧光显微镜观察到，此时各组细胞的转染效率均已达到90%以上，超过许多文献报道的质粒介导的RNA干扰的平均水平，说明了慢病毒介导的RNA干扰的高效性。在RNA干扰后7天，进行Western blot检测，可以观察到转染了GRP75 shRNA后的A549细胞和A549/CDDP细胞GRP75蛋白的表达均明显下调，说明慢病毒介导的RNA干扰能长时间稳定地沉默目的基因的表达，并且具有很强的特异性。同时我们也观察到，在转染了不带有干扰序列的慢病毒后，A549细胞和A549/CDDP细胞GRP75蛋白的表达不受影响，说明慢病毒本身并不抑制GRP75蛋白的表达。通过慢病毒介导的RNA干扰，我们成功地将GRP75 shRNA转染入A549细胞和A549/CDDP细胞，并且GRP75 shRNA有效地抑制了GRP75蛋白的表达。

在确认GRP75蛋白的表达被有效抑制后，我们对六组细胞进行了MTT检测，以观察它们在不同浓度的顺铂作用48 h后的存活情况。通过实验结果我们观察到，当GRP75蛋白的表达被抑制后，A549细胞和A549/CDDP细胞对顺铂的耐受性均降低，说明了GRP75蛋白是A549细胞对顺铂的耐药机制的相关蛋白。同时，我们观察到转染了不带干扰序列的慢病毒后，A549细胞和A549/CDDP细胞对顺铂的敏感性没有明显地改变，说明慢病毒本身并不影响A549细胞和A549/CDDP细胞对顺铂的敏感性。但是相对于A549/CDDP细胞耐药性的较大幅度降低，A549细胞的耐药性降低并不明显。并且我们观察到，虽然经过RNA干扰后，没有达到完全封闭GRP75表达的效果，但是耐药细胞中GRP75蛋白的表达水平已经低于敏感细胞，即使如此，A549/CDDP细胞对顺铂的敏感性仍明显地低于A549细胞。这些结果表明，GRP75是A549细胞对顺铂耐药机制的相关蛋白之一，通过抑制GRP75蛋白的表达可以一定程度上改善A549细胞对顺铂的耐药性。

GRP75是一种分布于线粒体和胞质内的重要的分子伴侣^[5]^，其N端1-23位点为线粒体的穿膜信号，可以帮助蛋白质正确折叠，协助蛋白质的转运。在细胞能量代谢过程中GRP75蛋白可以帮助与能量代谢相关的生物大分子穿过线粒体参与能量代谢的各种反应。在缺糖、辐射等应激条件下，GRP75表达上调，有提高细胞对应激反应耐受性的作用，对缺糖造成的细胞损伤也有明显的保护作用。Grp75还参与调节细胞内葡萄糖水平、调控细胞增殖和分化、处理抗原等生理过程^[6-8]^。另外还有研究^[9, 10]^显示，GRP75还有抑制细胞凋亡的作用，可能与其能修复受损蛋白的功能有关。顺铂的作用机理是进入肺癌细胞后，与DNA交叉联结，形成稳定的顺铂-DNA复合物，阻断DNA复制，引起肺癌细胞死亡，其中凋亡是主要死亡方式。由于GRP75主要定位于线粒体，因此它在对抗顺铂的过程中所起的作用，并不是直接参与了阻止顺铂进入细胞核，或者修复DNA损伤，更可能是通过抑制细胞凋亡，使细胞获得更多的修复时间，从而得以存活。

野生型p53是调控细胞凋亡的重要因子，其介导的细胞凋亡途径包括转录依赖性途径和非转录依赖性途径。转录依赖性途径是当细胞受损时，细胞内野生型p53表达上调，继而其下游基因，包括*Bax*、*p53Alpx*、*P21*、*mdm2*、*Fas*/*Apo-1*、*Noxa*、*PERP*、*DARL*、*PIDD*等^[11-13]^，发生表达，引起线粒体跨膜电位的改变，产生线粒体活性氧，加剧细胞的损伤，诱导细胞凋亡。非转录依赖性途径是指野生型p53不进入细胞核诱导上述基因转录，而是在细胞质中直接激活Caspase途径而启动细胞凋亡进程^[14, 15]^。本研究通过Western blot检测转染前后A549细胞和A549/CDDP细胞内野生型p53的表达。我们观察到，在转染前，对比A549细胞，A549/CDDP细胞野生型p53表达明显下调，提示A549细胞对顺铂耐药机制与野生型p53的下调有关。虽然在转染了不带干扰序列的慢病毒后，A549细胞和A549/CDDP细胞野生型p53表达均有略微上调，但是在转染了GRP75 shRNA后，A549细胞和A549/CDDP细胞的野生型p53表达出现非常显著地上调。以上结果表明，当GRP75的表达上调时，野生型p53的表达下调，而当GRP75的表达下调时，野生型p53的表达上调。国外有研究^[16]^证实，p53直接介导bax导致线粒体膜通透性改变和凋亡，而GRP75也是位于线粒体，因此我们认为GRP75在耐药机制中的作用与对野生型p53的调控有关。GRP75通过抑制野生型p53的表达，从而保护了被顺铂损伤的肿瘤细胞，避免其死亡。

bcl-2是抑制凋亡的重要因子，主要分布于线粒体膜上，调节线粒体的功能状态。bcl-2过表达可抑制c-myc诱导的细胞凋亡，可以抑制依赖p53的凋亡途径，还可以抑制非依赖p53的凋亡途径。bcl-2不阻止药物进入细胞内，不抑制药物造成的DNA损伤，也不加速细胞的修复。近年来的研究认为bcl-2可能通过清除线粒体活性氧^[17]^，增强细胞的抗氧化能力^[18]^，来保护细胞免于死亡。本研究通过Western blot检测转染前后A549细胞和A549/CDDP细胞bcl-2的表达。我们观察到，在转染前，对比A549细胞，A549/CDDP细胞bcl-2表达明显上调，提示bcl-2可能也是A549细胞对顺铂耐药机制的相关蛋白。当转染了GRP75 shRNA以后，A549/CDDP细胞的bcl-2表达明显下调。GRP75与bcl-2都是位于线粒体，两者可能通过调节线粒体的功能状态，对受顺铂损伤的肿瘤细胞有协同保护作用。

综上所述，我们通过慢病毒介导的RNA干扰，成功地将GRP75 shRNA转染入A549细胞和A549/CDDP细胞，有效地抑制了GRP75蛋白的表达，一定程度逆转了A549/CDDP细胞对顺铂的耐药性，同时观察到GRP75对肿瘤细胞耐药性的影响与p53和bcl-2有关，但是尚不能阐明具体机理，这仍需要进一步的深入研究。
